# Supplementary material for: Synergy of metal nanoparticles and organometallic complex in NAD(P)H regeneration via relay hydrogenation
Source: Nat Commun. 2022 Sep 28;13:5699. doi: 10.1038/s41467-022-33312-x (PMC9519545; doi:10.1038/s41467-022-33312-x)
Supplement: Supplementary file 1 — Supplementary information [file 41467_2022_33312_MOESM1_ESM.pdf]

Supplementary information

Synergy of metal nanoparticles and organometallic complex in NAD(P)H regeneration via relay hydrogenation

Maodi Wang[1,2], Zhenchao Zhao[3], Chunzhi Li[1,2], He Li[1], Jiali Liu [1,2] and Qihua Yang[3*]

[1]State Key Laboratory of Catalysis, Dalian Institute of Chemical Physics, Chinese Academy of Sciences, 457 Zhongshan Road, Dalian 116023, China

[2]University of Chinese Academy of Sciences, Beijing 100049, China

[3]Key Laboratory of the Ministry of Education for Advanced Catalysis Materials, Zhejiang Key Laboratory for Reactive Chemistry on Solid Surfaces, Institute of Physical Chemistry, Zhejiang Normal University, Jinhua 321004, China

*E-mail: yangqh@dicp.ac.cn

Supplementary methods.

Chemicals

All chemicals were of analytical grade and used as received without further purification. Ruthenium (III) chloride hydrate ($RuCl_3·xH_2O$), and sodium tetrachloropalladate (II) (Na_2PdCl_4) were obtained from InnoChem. Rhodium (III) chloride hydrate ($RhCl_3·xH_2O$), chloroplatinic acid ($H_2PtCl_6.xH_2O$) and β-Nicotinamide adenine dinucleotide phosphate sodium salt ($NADP^+$) were purchased from Macklin Chemicals. 1,3,5-Triformylphloroglucinol (TP) and 4,4',4''-(1,3,5-triazine-2,4,6-triyl)trianiline (TTA) were purchased from Jilin Province Extension Technology Co. KH_2PO_4, K_2HPO_4, glucose, 1,10-phenanthroline, and $Ni(NO_3)_2·6H_2O$ were purchased from Sinopharm Chemical Reagent Co. Ltd. $(Cp^*RhCl_2)_2$, $(Cp^*IrCl_2)_2$ and (hexamethylbenzene)ruthenium(II) dichloride dimer were purchased from Bidepharm. β-Nicotinamide adenine dinucleotide (NAD^+), β-nicotinamide adenine dinucleotide reduced disodium salt hydrate (NADH), propionaldehyde, 2,2'-bipyridine and TiO_2 (rutile, 25 nm) were purchased from Aladdin Reagent Company. Alcohol dehydrogenase (ADH, 1938 U/mL) and glucose dehydrogenase (GDH, 200 U/mg) were obtained from Yuanye Biology (Shanghai).

Characterization

Transmission electron microscopy (TEM) was performed on a HITACHI 7700 at an acceleration voltage of 100 kV. The HAADF-STEM images and STEM-EDX mappings were undertaken on a JEOL F200 instrument. The N_2 adsorption-desorption experiments were performed at 77 K using a Micromeritrics ASAP 2020. Samples were degassed at 120 °C for 6 h prior to the measurements. X-ray diffraction (XRD) patterns were recorded on a Rigaku RINTD/Max-2500 powder diffraction system equipped with Cu Kα radiation, scanning from 2° to 80° with a rate of 10 °/min. CO chemisorption measurement was carried out at 50 °C on Micrometrics Auto-Chem 2910 with a thermal conductivity detector (TCD). Each catalyst was pretreated at 200 °C under a flow of 10% H_2/N_2 atmosphere (30 mL/min) for 1 h. UV-vis spectra were recorded on SHIMADZU UV-vis 2550 spectrophotometer. FT-IR spectra in the range of 400 to 4000

cm^{-1} were collected with a Nicolet Nexus is50 IR spectrometer using KBr pellets. Transmission electron microscopy (TEM) was performed on a HITACHI 7700 at an acceleration voltage of 100 kV. In situ Fourier-transformed infrared (FT-IR) spectra of CO adsorption were collected on a Thermo iS50 infrared spectrometer with a mercury cadmium telluride (MCT) detector and a transmission cell. Before the measurement, the heterogenous catalysts were pretreated at 200 °C under a 10% H_2/N_2 flowing (30 mL/min) for 1 h. The catalysts were subsequently cooled down to room temperature under a flowing N_2 (30 mL/min). After evacuating for 30 min, the spectrum of background was collected. A pulse of CO was introduced to the catalyst at room temperature. X-ray photoelectron spectroscopy (XPS) experiments were tested on a Kratos AXIS HSi spectrometer equipped with a monochromated Al Kα source (1486.7 eV) and a charge neutralizer. ^{1}H NMR spectra were recorded on Bruker Avance 400 or 700 MHz spectrometer at room temperature. To eliminate the interference of deuterated reagent, D_2O was encapsulated in a capillary quartz tube, which was placed in the nuclear magnetic tube containing targeted sample.

Chemoenzymatic reduction of acetophenone.

Chemoenzymatic reduction of acetophenone was carried out in a stainless-steel autoclave (300 mL). To be specific, 9 mg Ni/TiO_2, **2** (0.268 μmol Rh), 20 μL AKR (72.7 mg mL^{-1}), acetophenone (11 mg) and PB (2 mL, pH = 7.0) containing $NADP^+$ (1.5 mM) were loaded into ampule tube. The system was purged with H_2 for 6 times to remove the air, then the reaction was carried at 37 °C, 2 MPa H_2. After the reaction, the mixture was extracted with ethyl ether (2 mL) for 3 times. The organic phase was analyzed by GC equipped with a Supelco β-Dex 225 chiral column to determine the conversion and ee.

Supplementary Figures and Tables.

Supplementary Table 1. Comparison of the results of NAD^+ hydrogenation in this work and in literatures.

Catalyst	NAD^+ (mM)	T (°C)	H_2 (atm)	pH	Sel. (%)	Specific activity (mol_{NAD^+} mol^{-1}_{metal} h^{-1})[a]	Ref.
Ru/TiO_2@10TP-TTA & **1**	1.5	37	20	8.7	>99	91.6	This work
	1.5	37	20	7.0	>99	90.7	
	1.5	37	1	8.7	>99	38.3	
	1.5	25	1	8.7	>99	13.6	
	15	37	20	9.4	92.0	47.2	
Pt/ana-450	1.5	37	1	8.7	63.4	19.9	1
Pt/SiO_2	1.5	22	9	7.0	30	~512	2
Pt90Sn10/SiO_2	0.1	22	9	7.0	~90	46.3	
[Ir(Cp*)(4-(1H-pyrazol-1-yl-κN^2)benzoic acid-κC^3)(H_2O)]$_2SO_4$	0.77	25	1	6.5	97[b]	36	3
	0.77	25	1	7.0	97[b]	~30	
	1.9	25	1	7.0	97[b]	<10	

[a]The specific activity was calculated based on all of the metals used in reactions. [b]Yield of NADH.

Supplementary Table 2. Physicochemical Parameters of Ru/TiO_2@TP-TTA

Catalyst	BET surface area (m^2/g)	w.t.% of TP-TTA[a]	Ru dispersion (%)[b]
Ru/TiO_2	24	-	12.2
Ru/TiO_2@5TP-TTA	42	3.0	6.7
Ru/TiO_2@10TP-TTA	71	7.1	2.9

[a]Data obtained from TG analysis. [b]Data derived from CO chemisportion.

Supplementary Table 3. Catalytic performance of NPs and **1** in NAD^+ hydrogenation[a].

Entry	Catalyst	Time (min)	Conv. (%)	Sel. (%)
1	**1**	30	16.5	94.0
2	Pt/TiO_2@5TP-TTA	30	100	67.8
3	Rh/TiO_2@5TP-TTA	30	97.8	15.3
4	Pd/TiO_2@5TP-TTA	30	87.2	51.5
5	Ru/TiO_2@5TP-TTA	30	67.4	45.9
6	Ru/TiO_2	30	23.9	48.5
7	Ru/TiO_2@10TP-TTA	30	20.8	66.3
8	Ni/TiO_2	40	4.7	n.d.
9	Ru/TiO_2 & **1**	20	100	85.9
10	Ru/TiO_2@5TP-TTA & **1**	5	100	95.6
11	Ru/TiO_2@10TP-TTA & **1**	8	100	>99
12	Rh/TiO_2@5TP-TTA & **1**	6	100	87.6
13	Rh/TiO_2@10TP-TTA & **1**	6	100	90.3
14	Pd/TiO_2@5TP-TTA & **1**	10	100	94.4
15	Pd/TiO_2@10TP-TTA & **1**	30	97.8	96.8
16	Pt/TiO_2@5TP-TTA & **1**	10	100	83.8
17	Pt/TiO_2@10TP-TTA & **1**	10	100	87.9
18	Ni/TiO_2 & **1**	40	100	95.1

[a]Reaction conditions: 0.29 μmol NPs and/or 0.067 μmol Rh, 1.5 mM NAD^+, 2 mL of 0.1 M phosphate buffer (PB, pH = 8.7), 37 °C and 2 MPa H_2.

Supplementary Table 4. Results of chemoenzymatic reduction of acetophenone.

NPs	Yield (μmol)	ee (%)
Ru/TiO_2@10TP-TTA	47.4	40.4
Rh/TiO_2@10TP-TTA	41.8	54.5
Pd/TiO_2@10TP-TTA	17.7	81.2
Pt/TiO_2@10TP-TTA	47.7	64.6
Ni/TiO_2	76.6	>99

Reaction conditions: 0.29 μmol metal NPs (3.1 μmol Ni) and 0.268 μmol Rh (**2**), 20 μL AKR, acetophenone (92 μmol), 3.0 μmol $NADP^+$, 2 mL of 0.1 M PB, pH = 7.0, 37 °C and 2 MPa H_2, 15h.

Noting: As a proof-of-concept, we further coupled in situ NADPH regeneration with a NADPH-dependent aldehyde ketone reductase (AKR) for the asymmetric reduction of acetophenone using $NADP^+$. Ru/TiO_2@10TP-TTA & **2** was firstly chosen as a model catalyst for in situ NADPH regeneration because of its high activity and NADH selectivity. However, only 40.4% ee was obtained for chemoenzymatic reduction of acetophenone because Ru/TiO_2@10TP-TTA can directly catalyze the acetophenone hydrogenation. The similar phenomenon was observed for other supported noble metal catalysts. In order to increase the ee value, Ni/TiO_2 was also tested in chemoenzymatic reduction of acetophenone since it shows no activity in acetophenone hydrogenation (Supplementary Fig. 19). After coupling with AKR, Ni/TiO_2 & **2** gave >99% ee, much higher than that of Ru/TiO_2@10TP-TTA & **2**, implying the importance of suppression of nonenzymatic acetophenone hydrogenation in ee promotion. AKR and **2** coupling system gave a phenyl ethanol yield of 20.0 μmol in 15 h. While a much higher amount (76.6 μmol) of phenyl ethanol was produced with AKR and Ni/TiO_2 & **2** coupling system. Furthermore, the regeneration efficiency was evaluated by turnover number (TON), which was defined as moles of product produced with per mole of $NADP^+$. The TON was calculated to be 24 for Ni/TiO_2 & **2**, indicating that NADPH could be regenerated for at least 24 cycles in this chemoenzymatic reduction system. The product yield and TON are superior to most of the reported artificial NAD(P)H regeneration systems (Supplementary Table 5), such as transfer hydrogenation, photocatalysis and electrocatalysis. The efficient NAD(P)H regeneration from $NAD(P)^+$ avoids the direct

consumption of stoichiometric amount of instable and expensive NAD(P)H. Our results highlight the great superiority of the application of bioreduction in vitro via in situ NAD(P)H regeneration using coupled metal NPs and metal complex catalysts.

Supplementary Table 5. Comparison of the results of chemoenzymatic reduction in this work and representative results in literatures.

NAD(P)H regeneration catalyst	Conditions	Time (h)	Amount of $NAD(P)^+$	Substrate	ee (%)	Product (μmol)	TON	Ref.
Ni/TiO_2 & **2**	37 °C, 2 MPa H_2	15	1.5 mM, 2 mL	acetophenone	>99	76.6	24	This work
$[(\eta^5\text{-}C_5Me_5)Rh(phen)Cl]^+$	37 °C, 0.1 M formate	24	1 mM, 1 mL	acetophenone	98	6.6	6.6	6
Rh@PMO	40 °C, 0.1 M formate	42	1.5 mM, 2 mL	4-phenyl-2-butanone	>98	17.2	5.7	7
CCGCMAQSP & **1**	Photocatalysis	50	1 mM, 3 mL	acetophenone	>99	12.6	4.2	8
a-MoS_x	Electrocatalysis	4	1 mM, 2 mL	benzaldehyde	-	17.4	8.7	9
TP-COF & **1**	Photocatalysis	0.2	2 mM, 3 mL	a-ketoglutarate	-	2.9	0.5	10

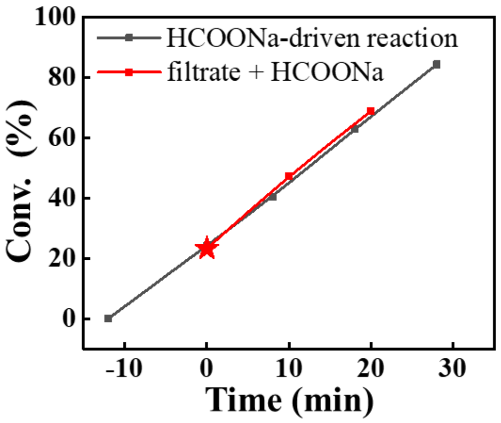

Supplementary Fig. 1 Reaction profiles of formate-driven reactions. Reaction profiles of formate-driven NADH regeneration catalyzed by **1** and the reaction profile of the filtration of Ru/TiO_2 & **1** with the addition of HCOONa. Reaction conditions: for the formate-driven reaction, **1** (0.033 mM), 1.5 mM NAD^+, 2 mL PB (pH = 8.7), 37 °C and 150 mM HCOONa; before the filtration, the reaction (3 mL PB containing 1.5 mM NAD^+) was catalyzed by Ru/TiO_2 (0.29 μmol Ru) and **1** (0.033 mM) under 2 MPa H_2 for 10 min, then Ru/TiO_2 was filtrated out and HCOONa was added in the filtration (2 mL) to drive the reaction.

Noting: It's acknowledged that the reaction order against NAD^+ is zero in the formate-driven NADH regeneration catalyzed by **1**[4], which was also demonstrated by the linearly increased conversion in our experiment. Thus, the conversion would not be sensitive to the concentration of NAD^+.

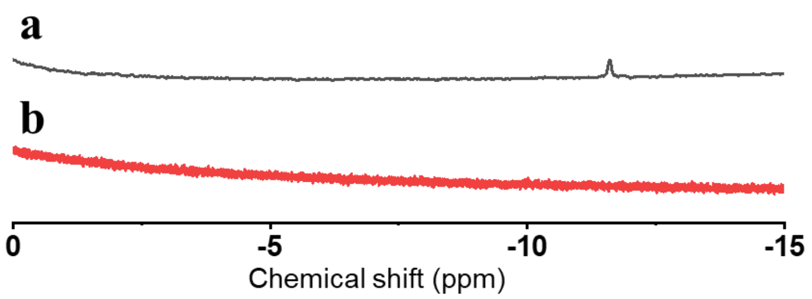

Supplementary Fig. 2 ¹H NMR spectra. ¹H NMR spectrum of **1** (21.5 mM) after incubation with (a) HCOONa (160 mM) overnight and (b) after H_2 treatment for 4 h in the presence of Ru/TiO_2. (700 MHz)

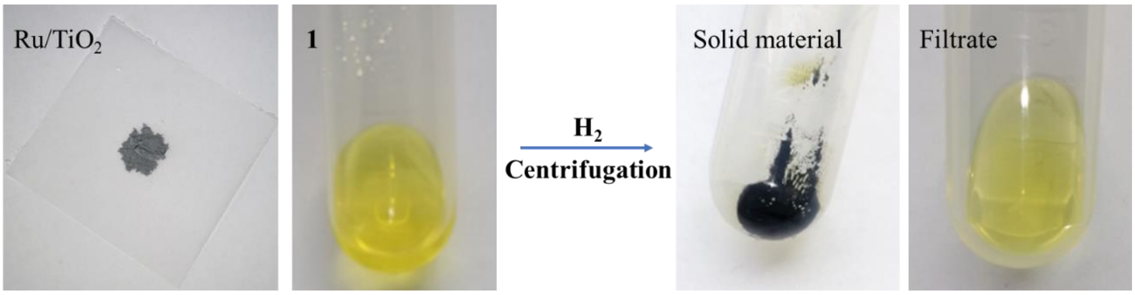

Supplementary Fig. 3 Photographs of Ru/TiO_2 & 1. Photographs of Ru/TiO_2 & **1** before and after treatment with H_2.

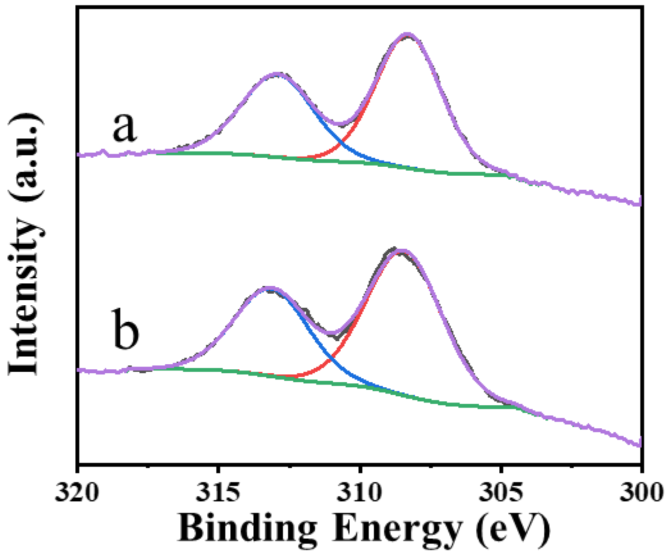

Supplementary Fig. 4 Rh 3*d* XPS spectra. Rh 3*d* XPS spectra of (a) **1** deposited on Ru/TiO_2 after H_2 treatment and (b) **1** after treatment with HCOONa.

For the analysis electronic states of Rh, the mixture of **1** and Ru/TiO_2 was treated with H_2, then the solid sample was separated by centrifugation and dried under vacuum for further analysis. In the formate-driven system, the solid sample was collected for analysis after removing of water under reduced pressure.

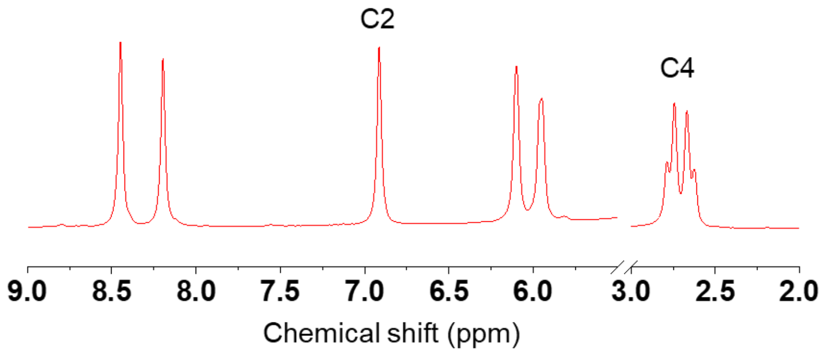

Supplementary Fig. 5 ^{1}H NMR spectrum of NADH. ^{1}H NMR spectrum of NADH after the pretreatment with Ru/TiO_2@10TP-TTA & **1** under D_2O/N_2 for 3 h. (400 MHz)

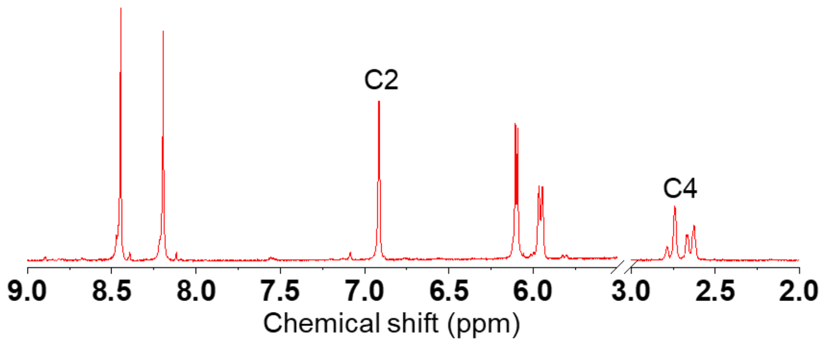

Supplementary Fig. 6 Isotope experiment using D_2O. ^{1}H NMR spectrum of the reaction solution from NAD^+ (15 mM) hydrogenation with Ru/TiO_2@10TP-TTA & **1** with D_2O/H_2. Reaction conditions: pH = 9.4, 37 °C and 2 MPa H_2. (400 MHz)

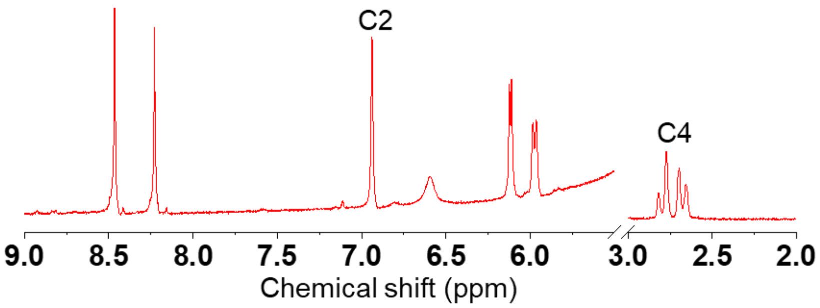

Supplementary Fig. 7 Isotope experiment using D_2. ^{1}H NMR spectrum of the reaction solution from NAD^+ (6 mM) hydrogenation with Ru/TiO_2@10TP-TTA & **1** with H_2O/D_2. Reaction conditions: pH = 8.7, 37 °C and 1 MPa D_2. (400 MHz)

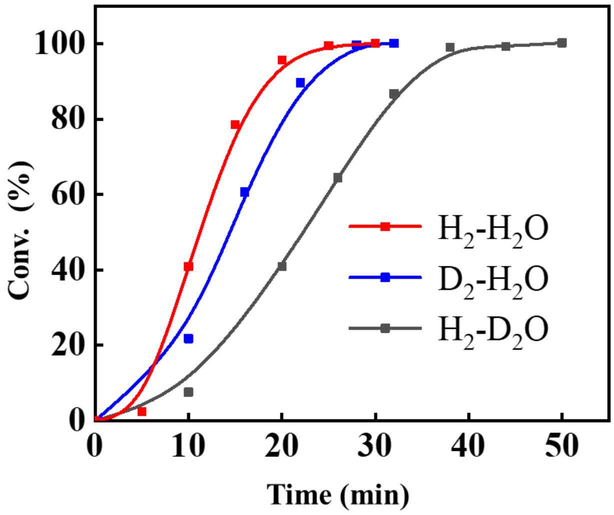

Supplementary Fig. 8 Kinetic isotopic experiments. Kinetic isotopic experiments catalyzed by Ru/TiO_2@10TP-TTA & **1**. Reaction conditions: (1.45 μmol Ru), (0.335 μmol Rh), 1.5 mM NAD^+, 10 mL of 0.1 M PB (pH = 8.7), 37 °C and 1 bar H_2/D_2.

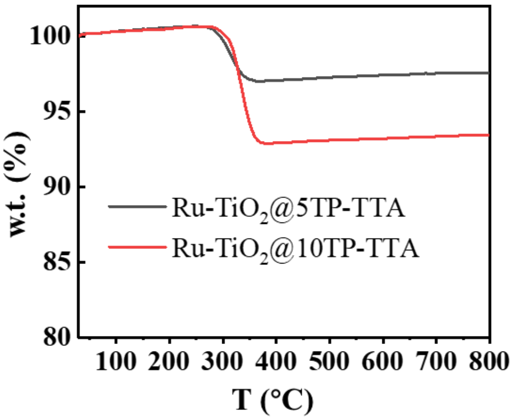

Supplementary Fig. 9 TG analysis. TG curve of Ru/TiO_2@TP-TTA.

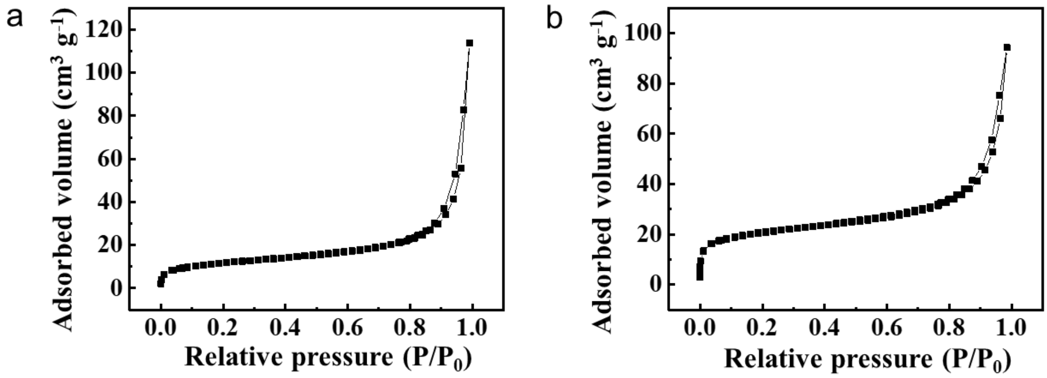

Supplementary Fig. 10 N_2 adsorption-desorption isotherm. N_2 adsorption-desorption isotherm of (a) Ru/TiO_2@5TP-TTA and (b) Ru/TiO_2@10TP-TTA.

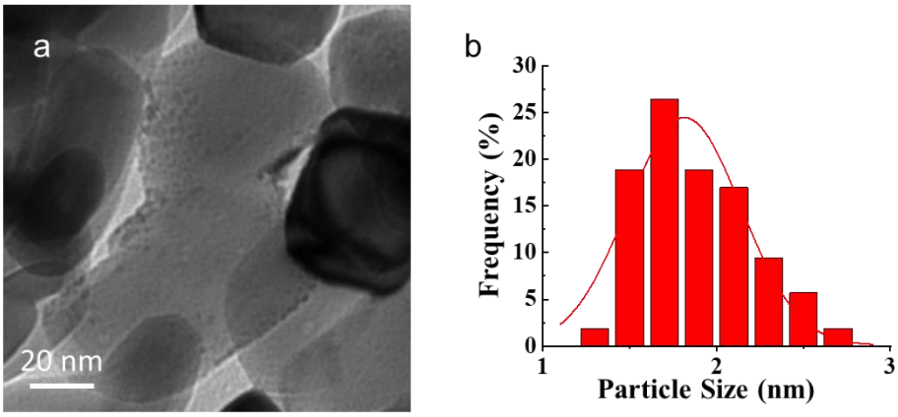

Supplementary Fig. 11 TEM images and the corresponding size distribution of Ru/TiO_2. TEM images of (a) Ru/TiO_2 and (b) the corresponding size distribution of Ru NPs.

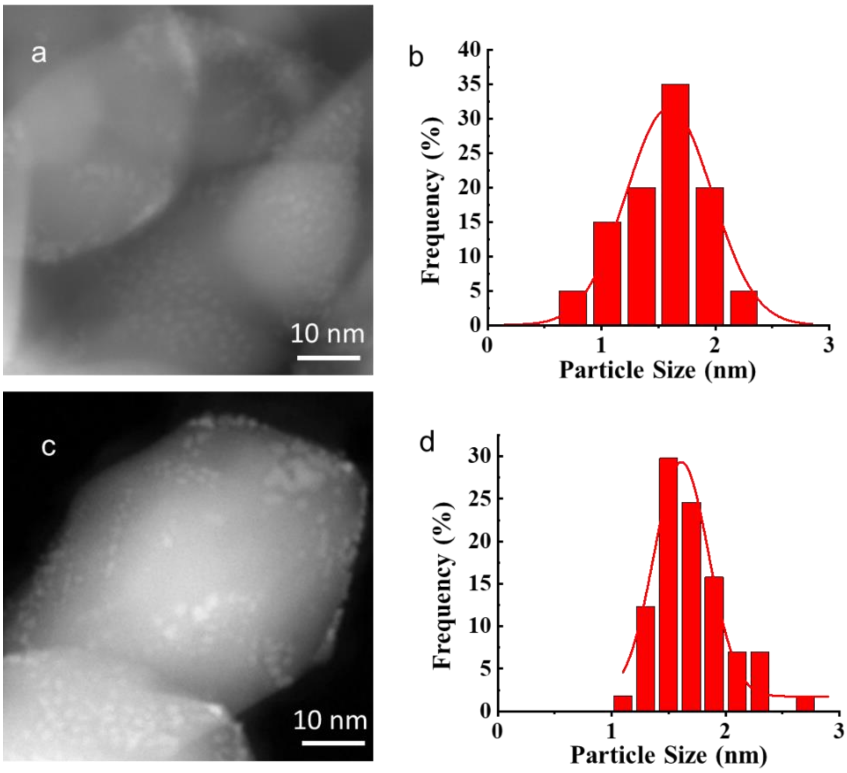

Supplementary Fig. 12 HAADF-STEM images and the corresponding Ru size distribution of Ru/TiO_2@TP-TTA. (a-b) Ru/TiO_2@5TP-TTA and (c-d) Ru/TiO_2@10TP-TTA.

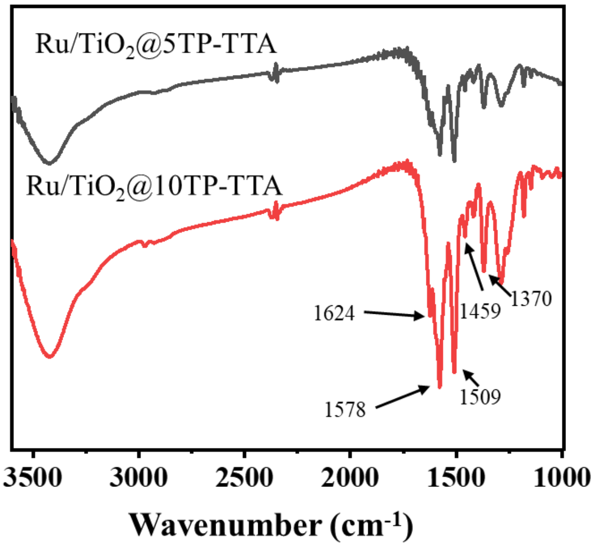

Supplementary Fig. 13 FT-IR characterization. FT-IR spectra of Ru/TiO_2@5TP-TTA and Ru/TiO_2@10TP-TTA.

Noting: The vibrations at 1624 cm^{-1}, 1578 cm^{-1} and 1509 cm^{-1} in the FT-IR spectra of Ru/TiO_2@TP-TTA respectively assigned to the vibrations from C=O, C=C and triazine ring indicate the formation of TP-TTA with β-ketoenamine linkage[5].

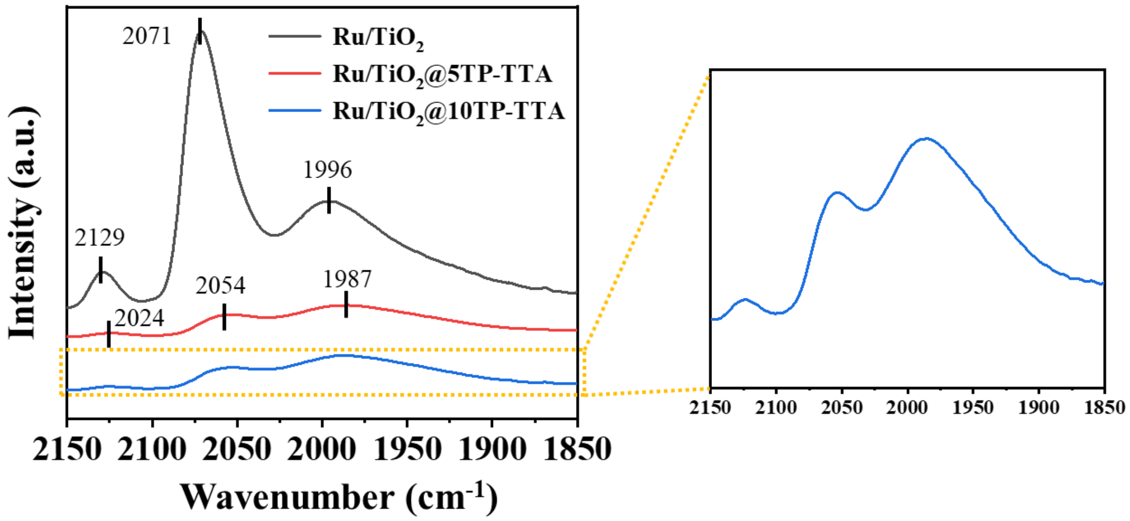

Supplementary Fig. 14 In situ FT-IR spectra of CO adsorption. In situ FT-IR spectra of CO adsorption of Ru/TiO_2@TP-TTA catalysts.

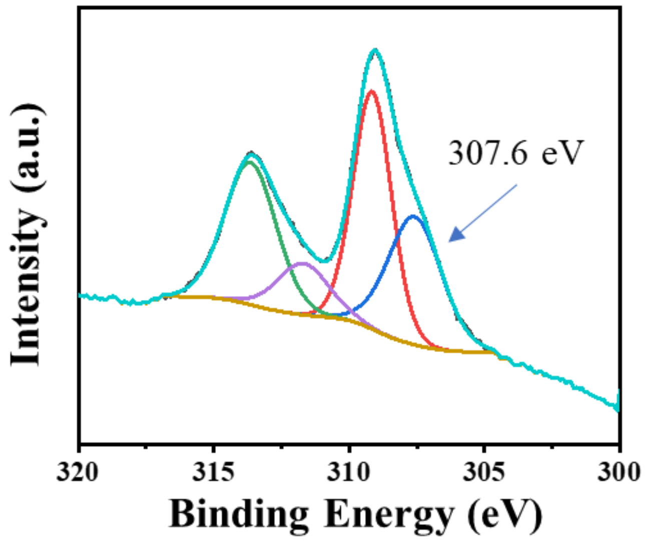

Supplementary Fig. 15 Rh 3*d* XPS spectra. Rh 3*d* XPS spectra of **1** after treatment with H_2.

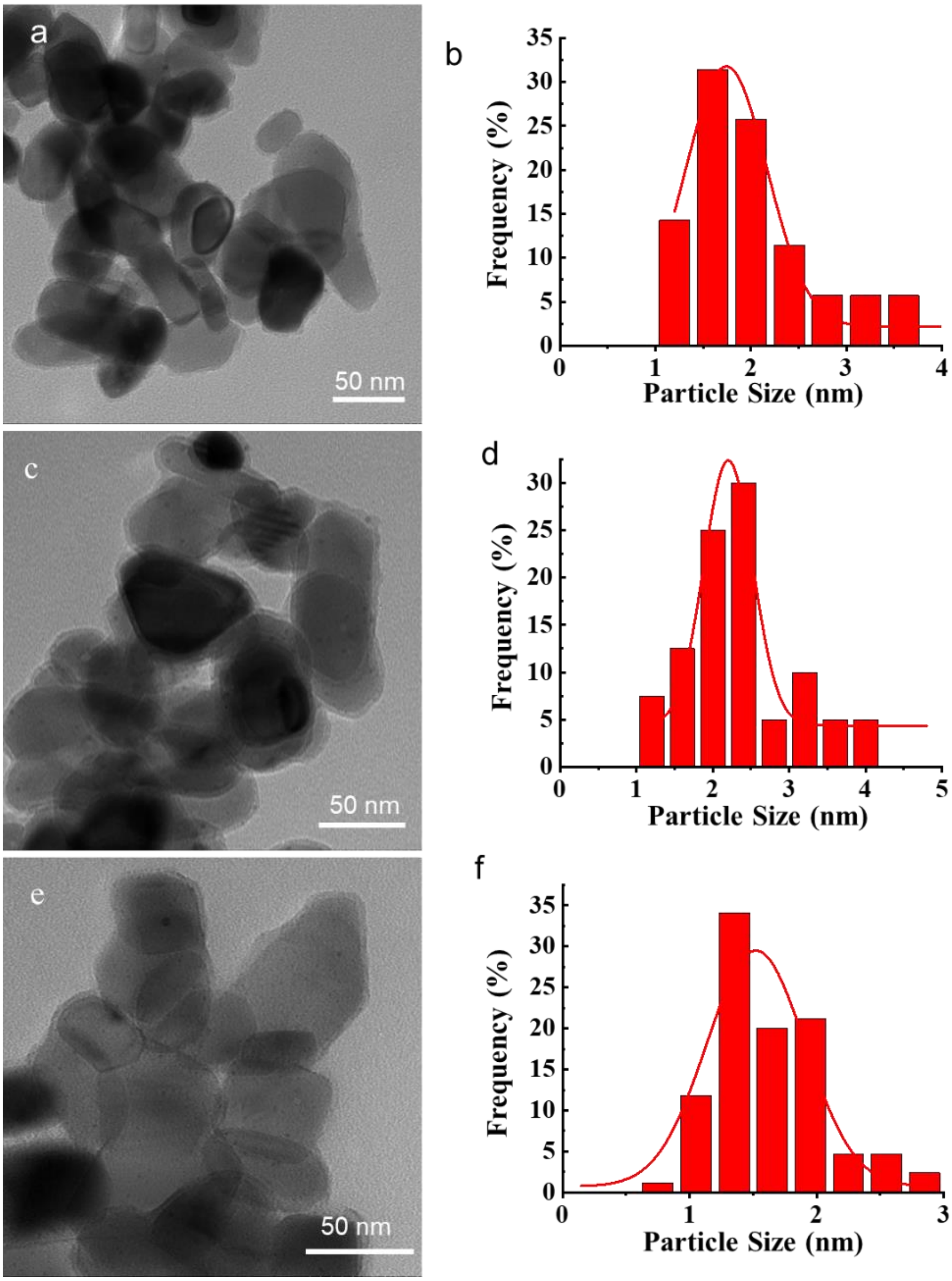

Supplementary Fig. 16 TEM images and the corresponding size distribution of metal NPs. (a, b) Rh/TiO_2@5TP-TTA, (c, d) Pd/TiO_2@5TP-TTA and (e, f) Pt/TiO_2@5TP-TTA.

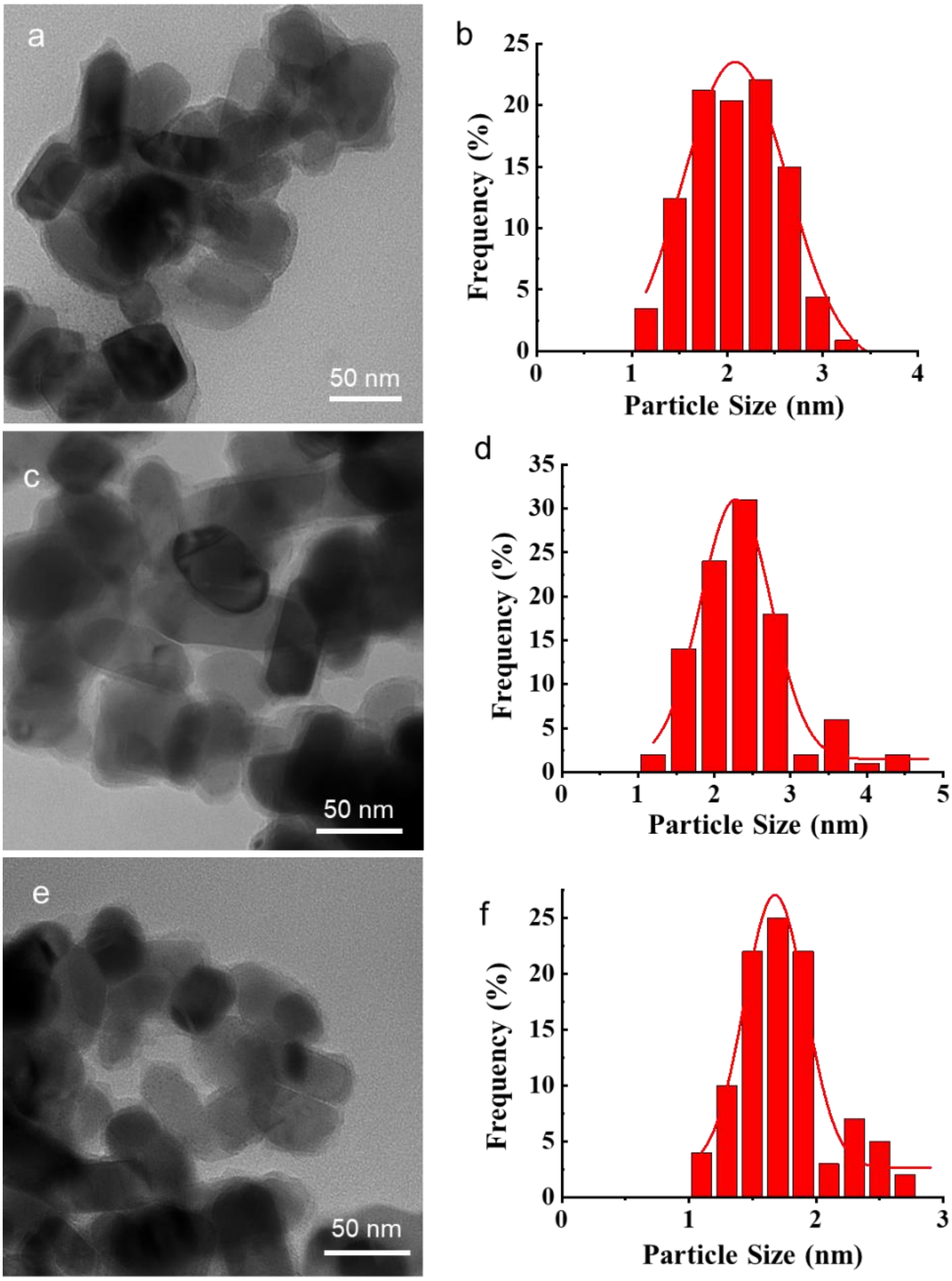

Supplementary Fig. 17 TEM images and the corresponding size distribution of metal NPs. (a, b) Rh/TiO_2@10TP-TTA, (c, d) Pd/TiO_2@10TP-TTA and (e, f) Pt/TiO_2@10TP-TTA.

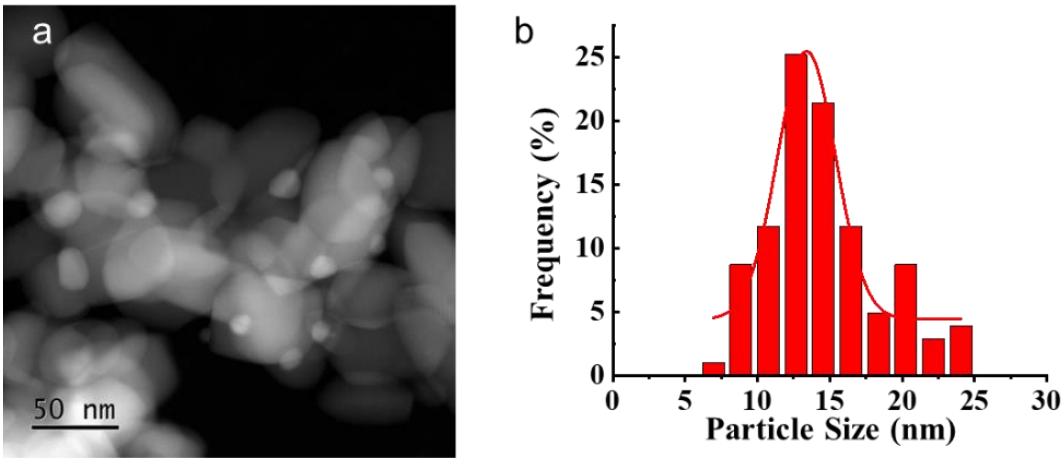

Supplementary Fig. 18 HAADF-STEM images and the corresponding Ni size distribution. (a) HAADF-STEM image and (b) the corresponding Ni size distribution of Ni/TiO_2.

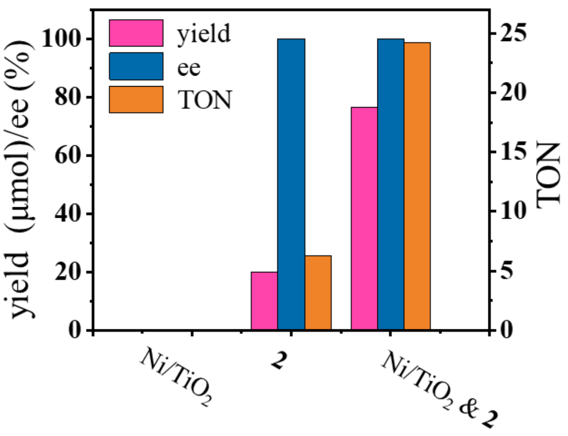

Supplementary Fig. 19 Chemoenzymatic reduction of acetophenone. Reaction conditions: 3.1 μmol Ni and/or 0.268 μmol Rh, 20 μL AKR, acetophenone (92 μmol), 3.0 μmol $NADP^+$, 2 mL of 0.1 M PB, pH = 7.0, 37 °C and 2 MPa H_2.

Supplementary references

1. Wang, M., *et al.* Chemoselective NADH regeneration: the synergy effect of TiO_x and Pt in NAD^+ hydrogenation. *ACS Sustain. Chem. Eng.* **9**, 6499-6506 (2021).
2. Burnett, J.W., *et al.* Directing the H_2-driven selective regeneration of NADH via Sn-doped Pt/SiO_2. *Green Chem.* **24**, 1451-1455 (2022).
3. Maenaka, Y., Suenobu, T. & Fukuzumi, S. Efficient catalytic interconversion between NADH and NAD^+ accompanied by generation and consumption of hydrogen with a water-soluble iridium complex at ambient pressure and temperature. *J. Am. Chem. Soc.* **134**, 367-374 (2012).
4. Hollmann, F., Witholt, B. & Schmid, A. $[Cp^*Rh\ (bpy)(H2O)]^{2+}$: a versatile tool for efficient and non-enzymatic regeneration of nicotinamide and flavin coenzymes. *J. Mol. Catal. B: Enzym.* **19**, 167-176 (2002).
5. Ren, X., *et al.* Enormous promotion of photocatalytic activity through the use of near-single layer covalent organic frameworks. *CCC Chem.* **3**, 2453-2463 (2021).
6. Canivet, J., Süss-Fink, G. & Štěpnička, P. Water-soluble phenanthroline complexes of rhodium, iridium and ruthenium for the regeneration of NADH in the enzymatic reduction of ketones. *Eur. J. Inorg. Chem.*, 4736-4742 (2007).
7. Himiyama, T., Waki, M., Maegawa, Y. & Inagaki, S. Cooperative catalysis of an alcohol dehydrogenase and rhodium-modified periodic mesoporous organosilica. *Angew. Chem. Int. Ed.* **131**, 9248-9252 (2019).
8. Choudhury, S., Baeg, J.O., Park, N.J. & Yadav, R.K. A photocatalyst/enzyme couple that uses solar energy in the asymmetric reduction of acetophenones. *Angew. Chem. Int. Ed.* **51**, 11624-11628 (2012).
9. Bau, J.A., *et al.* Mo^{3+} hydride as the common origin of H_2 evolution and selective NADH regeneration in molybdenum sulfide electrocatalysts. *Nat. Catal.* **5**, 397-404 (2022).
10. Zhao, Y., *et al.* Fully conjugated two-dimensional sp^2-carbon covalent organic frameworks as artificial photosystem I with high efficiency. *Angew. Chem. Int. Ed.* **131**, 5430-5435 (2019).
